# D_2_O as an Imperfect Replacement for H_2_O: Problem or Opportunity for Protein Research?

**DOI:** 10.1021/acs.jpcb.3c04385

**Published:** 2023-09-18

**Authors:** Giulia Giubertoni, Mischa Bonn, Sander Woutersen

**Affiliations:** † Van ’t Hoff Institute for Molecular Sciences, University of Amsterdam, Science Park 904, 1098XH Amsterdam, The Netherlands; ‡ Max Planck Institute for Polymer Research, Ackermannweg 10, 55128 Mainz, Germany

## Abstract

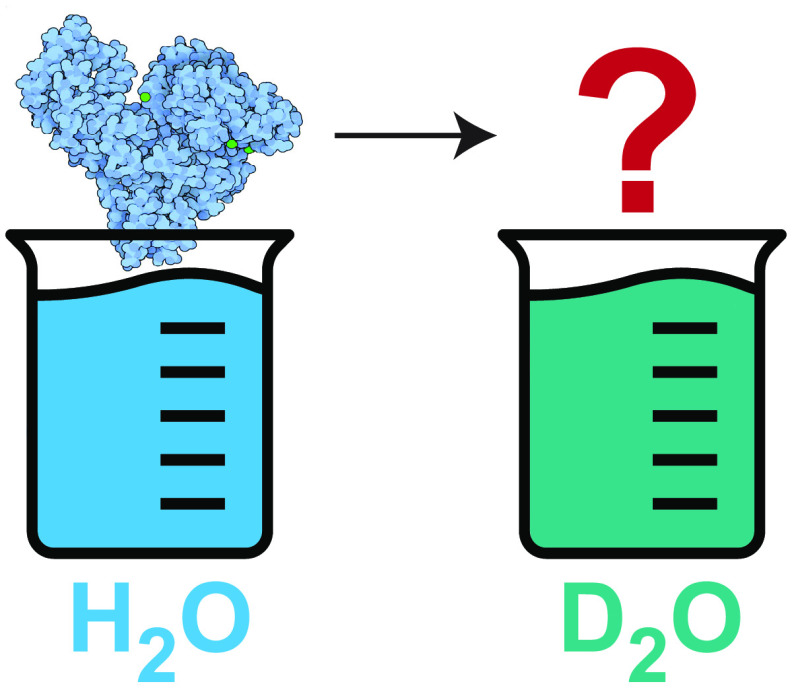

D_2_O is commonly used as a solvent instead of H_2_O in spectroscopic studies of proteins, in particular, in infrared and nuclear-magnetic-resonance spectroscopy. D_2_O is chemically equivalent to H_2_O, and the differences, particularly in hydrogen-bond strength, are often ignored. However, replacing solvent water with D_2_O can affect not only the kinetics but also the structure and stability of biomolecules. Recent experiments have shown that even the mesoscopic structures and the elastic properties of biomolecular assemblies, such as amyloids and protein networks, can be very different in D_2_O and H_2_O. We discuss these findings, which probably are just the tip of the iceberg, and which seem to call for obtaining a better understanding of the H_2_O/D_2_O-isotope effect on water–water and water–protein interactions. Such improved understanding may change the differences between H_2_O and D_2_O as biomolecular solvents from an elephant in the room to an opportunity for protein research.

## Introduction

D_2_O, or heavy water, is a stable isotopomer of H_2_O, containing deuterium instead of the most common hydrogen isotope protium. Deuterium was discovered in 1931 by H. Urey,^1^ who was awarded the Nobel Prize for this finding in 1934. The chemical and physical properties of D_2_O were first studied by G. Lewis and co-workers in the early 1930s^2,3^ and are very similar to those of H_2_O (Table 1). For this reason, D_2_O is often used as a solvent instead of H_2_O in experiments where the H atoms of water form a problem, such as in nuclear magnetic resonance, neutron scattering, and infrared spectroscopy and imaging. This holds in particular for studies of biomolecules: in both protein NMR and infrared spectroscopy and imaging,^4−6^ it is standard practice to use D_2_O as a solvent. In the case of infrared spectroscopy, this is done because the vibrational modes of the amide groups, which carry crucial information on the protein structure,^7^ have spectral overlap with the bending mode of H_2_O (both are in the 1600–1700 cm^–1^ frequency range). The D_2_O-bending frequency is 1250 cm^–1^, eliminating the overlap problem and making D_2_O the seemingly perfect replacement of H_2_O.

**Table 1:** Selected Physical and Chemical Properties of H_2_O and D_2_O^8−11^

property	H_2_O	D_2_O
molecular weight (g/mol)	18.02	20.03
melting point (°C)	0	3.82
boiling point (°C)	100	101.4
molar density (mol/L, 25.0 °C, 1 atm)	55.35	55.14
molecular polarizability (Å^3^)	1.45	1.26
viscosity (25 °C)	0.891	1.095
pH/pD (25 °C)	6.9976	7.43
dielectric constant (25 °C)	78.37	78.06

**Figure 1:**
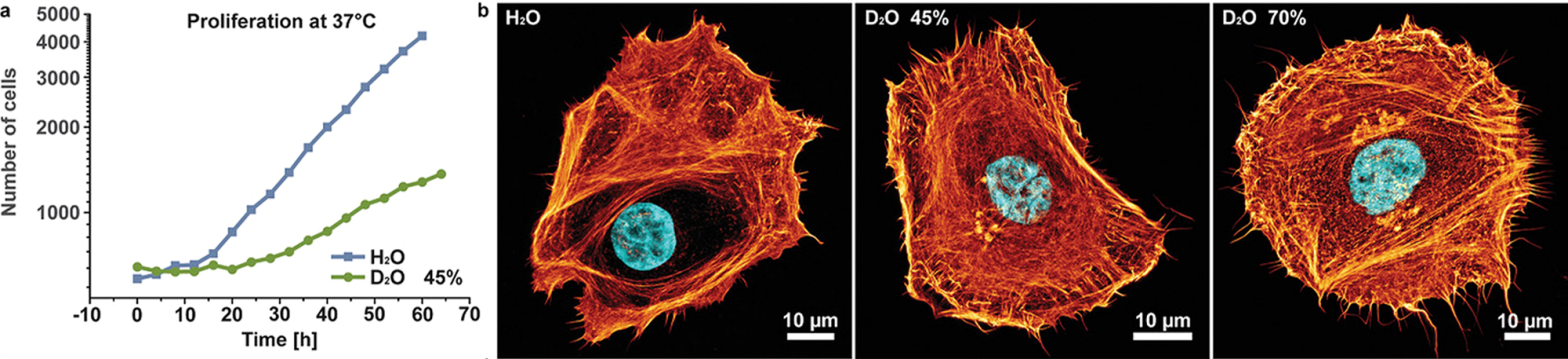
“Cells in slow motion.”^14^ (a) Epithelial cell proliferation is much slower in a D_2_O-rich medium. The duration of the cell cycle is roughly 17 h under normal (H_2_O) conditions and 44 h in water containing 45% D_2_O. (b) Independent of the culturing conditions, the actin cytoskeleton (orange) and the nucleus (blue) remain intact. Shown are typical pictures, which suggest that structures are comparable for the different H_2_O and D_2_O concentrations. Figure adapted from ref (14), Copyright 2021 Schnauß et al. (licensed under CC BY-NC 4.0).

The effect of H/D substitution on the kinetics of chemical reactions is well-known, and has been extensively studied and applied, for instance to study reaction mechanisms^12^ and to monitor protein folding.^13^ Interestingly, recent work shows that the kinetic effects induced by substituting D_2_O for H_2_O might also be useful for biomedical purposes:^14^ epithelial cells grown in a medium containing 45% D_2_O show significantly reduced migration and proliferation rates (Figure 1), and a similar slowdown in dynamics was observed in other cells,^15−17^ an effect that might find use for the storage of biological materials such as organs, or for anticancer treatment.^15^

While the effect of H/D substitution on kinetics is well established, it is often (implicitly) assumed that the effect of H_2_O/D_2_O substitution on the structure of biomolecules and biomolecular assemblies is small. However, although the use of isotopic substitution in spectroscopic experiments has been mostly successful, there is ample evidence that replacing H_2_O with D_2_O can alter the thermodynamic and structural properties of proteins^18−35^ and even the formation process and structure of protein assemblies.^14,25,33,36−41^ The fact that H_2_O/D_2_O replacement can change the structure of biopolymers and their assemblies should not come as a complete surprise: the hydrogen-bond^a^ structures of H_2_O and D_2_O are known to be different,^42^ and hydration and the hydrophobic effect are essential for all biomacromolecules, ranging from polysaccharides to proteins. Hydrating water molecules create a water network around solutes that not only acts as structure stabilizer but also mediates intra- and intermolecular interactions. As stated recently by Fischer et al.,^43^ hydration represents “an additional evolutionary constraint upon protein sequence to maintain ligand binding and modulate the affinity of those interactions”, to which we might add that since evolution has optimized protein structure and dynamics in H_2_O rather than D_2_O, and since the hydrogen-bond structures of these two liquids are different, differences in structure and dynamics are to be expected when replacing one with the other.

The H_2_O/D_2_O-induced changes in biomolecular structure seem to call for more detailed studies of the difference between liquid D_2_O and H_2_O, but they also suggest fascinating new research opportunities. In this Perspective, we first briefly describe the differences between H_2_O and D_2_O; then we summarize and discuss the existing experimental evidence for isotope-induced structural changes in biomolecules and biomolecular assemblies; finally, we discuss the current challenges and perspectives, in particular the possibility of using D_2_O to investigate the role of hydration in protein stability and interactions.

## H_2_O versus D_2_O

The interplay of nuclear quantum effects (NQEs) underlying the physical and chemical differences between liquid D_2_O and H_2_O is quite subtle. Simply put, the low mass of the hydrogen atom makes it behave more as a delocalized quantum particle than the heavier deuterium. This delocalization can have a substantial effect on the hydrogen bond strength.^10^ Specifically, for an O–H···O hydrogen bond, the hydrogen-bond strength is a function of the O···O distance (the shorter, the stronger) and the O–H···O bond angle (the straighter, the stronger). The larger distance spread for H vs D leads to a strengthening of the H-bond, while the larger angular spread leads to a weakening. Hence, these two nuclear quantum effects have contrary consequences for the H-bond strength. Depending on the details of the H-bond, one or the other effect may dominate, resulting in a weakening or strengthening of H-bonds upon isotopic substitution. Short hydrogen bonds are typically strengthened due to NQEs, whereas long ones are weakened.^10^ Here we summarize the most important differences that are generally agreed upon in the literature, focusing on the points that are relevant for understanding how replacing H_2_O with D_2_O can change the structures of biomolecules and biomolecular assemblies.

**Figure 2:**
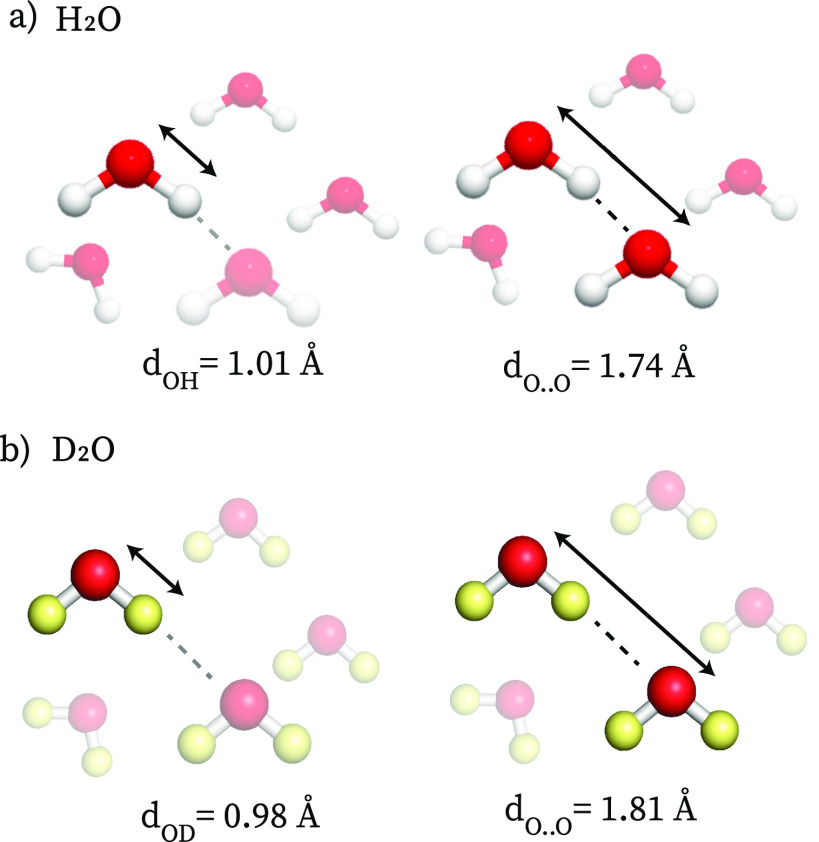
Average lengths of the covalent and hydrogen bonds in liquid H_2_O (a) and D_2_O (b).

The structure of liquid D_2_O and water has been investigated using different methods, in particular X-ray, γ-ray, and neutron scattering. By combining X-ray measurements with molecular simulations, it was found that the covalent bond between oxygen and protium (O–H) is 3% longer with respect to the one between oxygen and deuterium (O–D), see Figure 2 (neutron scattering studies indicate a somewhat smaller isotope effect on the covalent bond length^10^). In D_2_O, the hydrogen-bond network is more tetrahedral than that in H_2_O and the hydrogen-bond coordination number is higher,^42^ both effects indicating stronger hydrogen bonds and a more structured hydrogen-bond network. The average hydrogen-bond distance (the O···O distance of two hydrogen-bonded water molecules) is 4% longer in D_2_O, as is also reflected in its lower molar density compared to H_2_O (cf. the situation in ice, where the hydrogen bonds are also stronger than in liquid water). In *ab initio* calculations on hydrogen-bonded oligomers, it was also found that the hydrogen-bond strength is 0.2–0.3 kcal/mol larger in D_2_O than in H_2_O.^44^ Finally, the macroscopic thermodynamical properties (such as the specific heat and the melting point) of H_2_O and D_2_O also indicate stronger hydrogen bonding between D_2_O molecules, with a difference in hydrogen-bond energy similar to that found in the *ab initio* calculations.^10,45^

## Isotope-Induced Effects on Biomolecular Structure

We will now discuss examples of how the stronger hydrogen bonding in D_2_O can influence biomolecular structure and stability. First, we discuss the effects on individual biomolecules and then the more recently discovered D_2_O-induced effects on protein assemblies.

### Effects of Replacing H_2_O with D_2_O on Protein Stability, Structure, and Hydration

D_2_O-induced changes in protein stability depend in a complicated manner on changes in the (local) hydration, with both enthalpic and entropic contributions. Yet, the simple argument that the stronger hydrogen bonding between D_2_O molecules suppresses protein unfolding, favoring compact, folded proteins with minimal hydration seems to be sound. In Table 2, we give an overview of experimental results demonstrating the effect of D_2_O on biomolecular stability, structure, and rigidity, based on (and somewhat extending) the excellent overview given in ref (26). Most studies focus on the conformational stability in H_2_O and D_2_O. This is motivated by the potential use of D_2_O as a way to slow down thermal degradation, especially in pharmaceutical applications. Several studies have shown that the native or folded states of globular proteins such as bovine serum albumin (BSA), lysozyme, and tubulin are more stable in D_2_O than in H_2_O.^18−21^ For instance, using differential scanning calorimetry (DSC), it was found that the denaturation temperature of lysozyme and BSA is 2–3 °C higher in heavy water than in water.^18^ Circular dichroism (CD) experiments, which are more structure-sensitive than DSC measurements, showed that the onset temperature of the irreversible thermal denaturation (i.e., the temperature of the irreversible change of the secondary structure) of BSA is 58 °C in D_2_O while it is 50 °C in H_2_O.^19^ Upon heat-treatment at 65 °C, BSA also retains a larger percentage of monomers in heavy water than in water (85% versus 75%, respectively), again indicating that the BSA monomeric form is more stable in D_2_O.^20^ Similar results have been found for other, nonglobular proteins, such as acyl carrier proteins,^22^ collagen,^24^ ribonuclease A^27^ and *Drosophila* signal-transduction protein Drk.^26^ Similar enhanced stability of the folded state was also observed for κ-carrageenan, which undergoes to a liquid-to-gel transition by forming double helices, that are stabilized significantly more in D_2_O.^25^

**Table 2:** Effects of D_2_O on the Properties of Proteins and Other Biomolecules^a^

biomolecule	method	effect
bovine serum albumin^18^	DSC	enhanced stability of the native state, *T*_d_^D_2_O^ – *T*_d_^H_2_O^ ≈ 2–3 °C
bovine serum albumin^19^	CD	enhanced stability of the native state Irr. *T*_d_^D_2_O^ – Irr. *T*_d_^H_2_O^ ≈ 8 °C
bovine serum albumin^20^	DLS, Fl, UV–vis, SE-HPLC	enhanced stability of the native state monomer % at 65 °C: 85% in D_2_O, 75% in H_2_O
lysozyme^18^	DSC	enhanced stability of the native state, *T*_d_^D_2_O^ – *T*_d_^H_2_O^ ≈ 2–3 °C
tubulin^21^	CD, DSC, Fl	enhanced stability of the native state, *T*_d_^D_2_O^ – *T*_d_^H_2_O^ ≈ 3 °C
acyl carrier proteins^22^	NMR	enhanced stability of the native state, Δ*G*_N→U_^D_2_O^ = 2.3 kcal/mol; Δ*G*_N→U_^H_2_O^ = 1.8 kcal/mol
collagen peptides^24^	CD, DSC	enhanced stability of the folded state, *T*_m_^D_2_O^ – *T*_m_^H_2_O^ ≈ 4 °C
ribonuclease A^27^	DSC	enhanced stability of the native state, *T*_m_^D_2_O^ – *T*_m_^H_2_O^ ≈ 4 °C
*Drosophila* signal transduction protein^26^	NMR	enhanced stability of the folded state, *T*_m_^D_2_O^ – *T*_m_^H_2_O^ ≈ 12 °C
κ-carragenean^25^	DSC	enhanced stability of the folded state, *T*_gel→liq_^D_2_O^ – *T*_gel→liq_^H_2_O^ ≈ 3 °C
elastin-like peptides^28^	DSC, CD, IR	enhanced stability of the collapsed state, Propensity to form β-turn/β-aggregate, LCST^H_2_O^ – LCST^D_2_O^ ≈ 2–5 °C
peptides containing alanine^29^	CD	propensity for PPII structure: 5–200% higher PPII signal in D_2_O
plastocyanin^32^	MD	altered solvent–protein interactions: 10–30% reduction of protein–water H-bonds
test polypeptides^34^	MD	altered solvent–protein interactions
agarose (Ag2)^33^	NMR	lower solvent–polysaccharide affinity, *N*_w_^H_2_O^/*N*_w_^D_2_O^ ≈ 3.8
ribonuclease T1^31^	luminescence	increased protein rigidity, IPL^D_2_O^ = 36 ms, IPL^H_2_O^ = 28 ms
β-lactoglobulin^31^	luminescence	increased protein rigidity, IPL^D_2_O^ = 44 ms, IPL^H_2_O^ = 30 ms
liver alcohol dehydrogenase^31^	luminescence	increased protein rigidity IPL^D_2_O^ = 819 ms, IPL^H_2_O^ = 630 ms
alkaline phosphatase^31^	luminescence	increased protein rigidity, IPL^D_2_O^ = 2142 ms, IPL^H_2_O^ = 2060 ms
apo-azurin^31^	luminescence	increased protein rigidity IPL^D_2_O^ = 603 ms, IPL^H_2_O^ = 564 ms
TAS1R2/TAS1R3 receptor^30^	MD	smaller radius of gyration *R*_g_^D_2_O^ is ≈3% smaller than *R*_g_^H_2_O^
azurin,^35^ lactoglobulin, ribonuclease	MD	smaller radius of gyration *R*_g_^D_2_O^ is ≈1% smaller than *R*_g_^H_2_O^

a Abbreviations: *T*_d_ = denaturation temperature; Irr. *T*_d_ = irreversible denaturation temperature; *T*_m_ = melting temperature of the native state; *T*_0_ = transition temperature from folded-to-unfolded; *R*_g_ = radius of gyration; IPL = intrinsic Trp phosphorescence lifetime; Δ*G*_N→U_ = Gibbs energy of unfolding; *T*_gel→liq_ = gel-to-liquid transition temperature; LCST = lower critical solution temperature; *N*_w_ = number of hydration waters per mass unit of agarose; DSC = differential scanning calorimetry; SE-HPLC = size exclusion high-performance liquid chromatography; CD = circular dichroism; DLS = dynamic light scattering; Fl = fluorescence measurements; NMR = nuclear magnetic resonance; MD = molecular dynamics simulations.

Part of this table is taken from ref (26).

The increased stability of folded and native structures in D_2_O indicates a stronger tendency to adopt a more compact, less solvent-exposed conformation in this solvent. For instance, a D_2_O-induced tightening of the helical structure has been proposed for actin, based on combined rheological and fluorescence experiments.^14^ Similarly, Cremer et al. have shown that elastin-like polypeptides (ELPs) undergo a hydrophobic collapse that is accompanied by the formation of β-turn structures, which are significantly more stable in D_2_O.^28^ Increased stability of intermolecular β-sheet structures in D_2_O has been suggested for insulin dimers because of the 2-fold slower assembly kinetics in heavy water with respect to water, as observed with infrared and two-dimensional infrared spectroscopy, and because of a larger fraction of dimer in D_2_O than H_2_O in the initial structures as revealed by molecular simulations based on solution-phase small-angle X-ray scattering experiments.^39^ This again suggests a general preference for a more compact conformation in D_2_O. Moreover, specific secondary structures can be enhanced when proteins are dissolved in D_2_O. Circular-dichroism studies by Chellgren et al. have demonstrated that peptides containing alanine have a stronger propensity to form polyproline II (PP II) structure in D_2_O than in H_2_O.^29^ Since it is believed that the PP II conformation perturbs the bulk hydrogen-bond network of the surrounding water less strongly than does an α-helical conformation, this effect was attributed to the increased energetic cost of protein solvation in D_2_O.

The difference in protein stability and the preference for PP II structure suggest that interactions between solvent and protein might be modified in D_2_O compared to H_2_O, leading to changes in the intraprotein hydrogen-bond network. This possibility has been investigated mostly by means of molecular dynamics simulations of various biomolecules, such as plastocyanin,^32^ RNA hairpins,^23^ and peptides.^34^ Interestingly, in ref (32), it was observed that a reduction of the number of hydrogen bonds between solvent and protein occurs mostly when polar and positively charged side groups are involved, while the opposite is observed for negatively charged side groups. Overall, however, a 10–30% reduction in the number of water molecules engaged in hydrogen bonds with the protein was observed in D_2_O compared to H_2_O, which was correlated to the enhancement of intramolecular interactions in this solvent.^32^ A lower affinity between D_2_O and solute was also observed in NMR studies on agarose.^33^ The increased rigidity which Cioni et al. have observed for different proteins (see Table 2) also supports the idea that protein–solvent interactions are altered in D_2_O:^31^ using luminescence methods it was found for 5 proteins out of the 7 analyzed that D_2_O increases protein rigidity, with a protein-dependent rigidity enhancement. In this respect it is interesting to note that some proteins crystallize more efficiently in D_2_O than in H_2_O,^46^ a phenomenon that in the case of ref (46) was even accompanied by a difference in crystal symmetry and structure (whereas in general protein crystal structures seem to be independent of whether H_2_O or D_2_O is used^47−49^). The D_2_O-induced damping of conformational fluctuations can be attributed to stronger solvent–solvent interactions,^31^ which reduce protein hydration and promote intramolecular interactions (as was observed in ref (32)). The reduction in structural fluctuations in D_2_O may thus be explained by the fact that water–protein interactions can destabilize proteins by lowering the free-energy barriers between different conformations.

**Figure 3:**
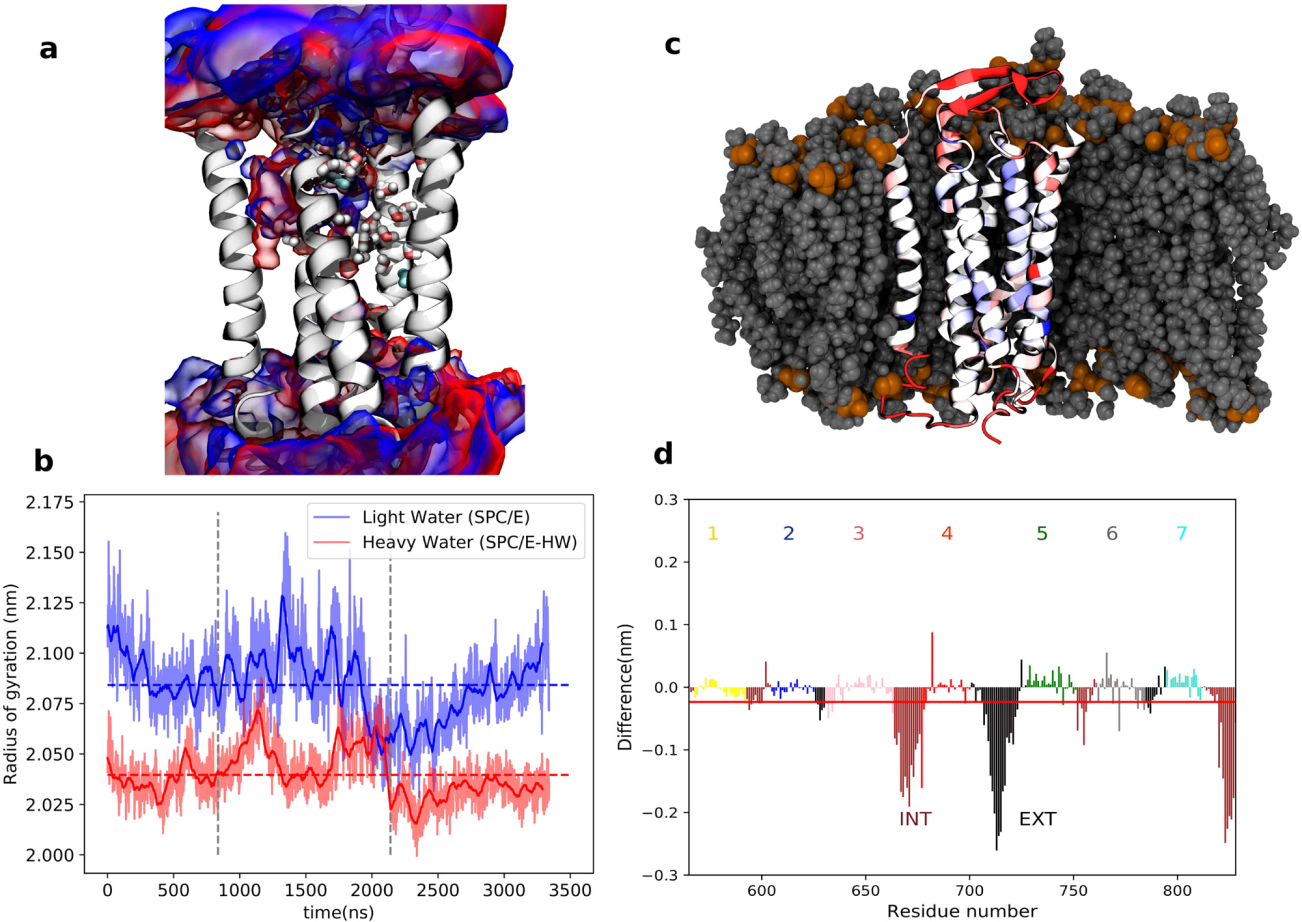
Differences between the behavior of the transmembrane part of the human sweet taste receptor in H_2_O vs D_2_O. (a) Structure of the TMD of the TAS1R2/TAS1R3 receptor with the probability density (volumetric map) of H_2_O (blue) or D_2_O (red) molecules within 10 Å of the protein. The conserved water molecules in the X-ray templates are shown in cyan. Water molecules predicted with the software OpenEye52 are shown in licorice representation. (b) Time evolution of the radii of gyration in H_2_O (blue) and D_2_O (red) from three microsecond time scale simulations (separated by vertical dashed lines) with total mean values as dashed lines, showing that the protein is more compact in D_2_O. (c) Snapshot of the transmembrane part of the human sweet taste receptor color-coded that red/blue represents parts more/less rigid in D_2_O vs H_2_O. The embedding lipid membrane is represented in gray. (d) Difference in root-mean-square fluctuations in MD trajectories. Negative/positive values mean that structures are more/less rigid in D_2_O than in H_2_O. The red line represents the sum of all residues. INT, intracellular; EXT, extracellular. Adapted from ref (30), copyright 2021 Ben Abu et al. (licensed under CC BY).

We conclude our list of proteins with the well-known and intriguing fact that D_2_O tastes sweet. A recent molecular-dynamics study of this isotope effect by the Jungwirth group^30^ has shown that the transmembrane part of the human sweet-taste sensor protein is more compact, stiffer, and subject to less structural fluctuations in D_2_O than in H_2_O (Figure 3). This study again supports the idea of a reduction in protein hydration in D_2_O compared to H_2_O. Indeed, in a more recent study the same group has found that in D_2_O, water has a stronger propensity to form water/water hydrogen bonds than water/amino-acid hydrogen bonds (interestingly, this behavior does not follow the hydrophobicity scale of the amino acids).^35^ It was also found that globular proteins (azurin, lactoglobulin, and ribonuclease) are significantly more compact in D_2_O than in H_2_O. Jungwirth et al. conclude that “D_2_O is a somewhat worse solvent for biomolecules than H_2_O. This also implies that association between proteins or between a protein and a biomembrane may be positively affected by water deuteration”. In the next section, we will see experimental results that support this idea.

### D_2_O-Induced Changes in Protein Assemblies and Networks

We have seen that D_2_O increases the stability of the folded state of proteins, in particular promoting the formation of secondary structures that least disrupt the hydrogen-bond network of water, and that protein hydration is reduced in D_2_O. More recently, it has become clear that these changes at the molecular level can affect the propensity and mechanisms of aggregation/assembly of biopolymers into larger supramolecular structures, leading to different mechanical and thermodynamic properties of the final aggregate/assembly (Table 3). In particular, Salvatella et al. have found that androgen receptors have a stronger tendency to form biomolecular condensates by liquid–liquid phase separation (LLPS) in D_2_O than in H_2_O.^36^ Interestingly, in this study, it was shown that replacing less than 10% water (as is common in NMR) with D_2_O can already significantly affect the phase equilibrium of the condensation, with a decrease of the cloud point by 0.5 °C for each added percent of D_2_O, and that the size of the condensates becomes larger with increasing amount of added D_2_O. These changes were attributed to the enhancement in D_2_O of the intermolecular interactions that drive the initial oligomerization. Similarly, an elegant study by Beckett et al. has shown that the dimerization of the *Escherichia coli* protein BirA is more favorable in D_2_O than in H_2_O, with a dimer dissociation constant that is 10 times smaller in the former.^50^ A similar D_2_O-induced alteration of the aggregation propensity (and possibly the final aggregate size) has been proposed for BSA aggregates, based on thioflavin fluorescence, turbidity, and circular dichroism experiments.^19,20,51^

**Table 3:** Effects of D_2_O on Biomolecular Self-Assembly^a^

protein	method	effect
*Escherichia coli* protein BirA^50^	SE	increased binding energy, *K*_dim_^H_2_O^/*K*_dim_^D_2_O^ ≈ 10
androgen receptor^36^	NMR, DLS, microscopy	enhanced condensation, larger condensates 25 °C shift of cloud point at a H_2_O/D_2_O fraction of 1:1
κ-carrageenan^25^	rheology	faster assembly, higher elastic modulus, *G*^′D_2_O^/*G*^′H_2_O^ ≈ 1.1–1.2
gelatin^37^	U-tube, rheology	faster assembly, higher shear modulus, *r*^D_2_O^/*r*^H_2_O^ ≈ 2.5, *G*^D_2_O^/*G*^H_2_O^ ≈ 3
casein^38^^b^	rheology	faster assembly, higher elastic modulus: Gel.On._RG_^D_2_O^ = 9.1 ± 0.1 min; Gel.On._RG_^H_2_O^ = 14.6 ± 0.1 min; Gel.On._TG_^D_2_O^ = 1.3 ± 0.4 min; Gel.On._TG_^H_2_O^ = 11.3 ± 1.1 min; *G*_RG_^′D_2_O^ = 1636.7 ± 75.7 Pa; *G*_RG_^′H_2_O^ = 1183 ± 55.1 Pa; *G*_TG_^′D_2_O^ = 504 ± 27.7 Pa; *G*_TG_^′H_2_O^ = 210 ± 26 Pa
insulin^39^	2DIR, IR, Fl	slower assembly, τ_lag_^H_2_O^ ≈ 16 h; τ_lag_^D_2_O^ ≈ 20 h
α-synuclein^40^	Fl, NMR, SANS	faster assembly, τ_lag_^H_2_O^ ≈ 34 h; τ_lag_^D_2_O^ ≈ 23 h (0.150 M NaCl)
actin^52^	static light scattering	formation of multifilament bundles in D_2_O, DCR^D_2_O(70%)^/DCR^H_2_O^ ≈ 2.5
agarose^33^	turbidity	change in the network, τ^D_2_O^/τ^H_2_O^ ≈ 1.1–1.3
pectin^41^	SAXS	change in network fractal dimension

a Abbreviations: *K*_dim_ = equilibrium dissociation constant for dimerization; τ_lag_= lag time; *G*′ = elastic modulus at a frequency of 1 Hz; *r* = rate of initial gelation; *G* = shear modulus; DCR = derived count rate (light-scattering intensity); Gel.On. = gelation onset; τ = initial turbidity; SE = sedimentation equilibrium measurements; 2DIR = two-dimensional infrared spectroscopy; SAXS= small angle X-ray scattering; SANS = small-angle neutron scattering.

b Two methods were used to induce gelation, referred to as RG and TG.

**Figure 4:**
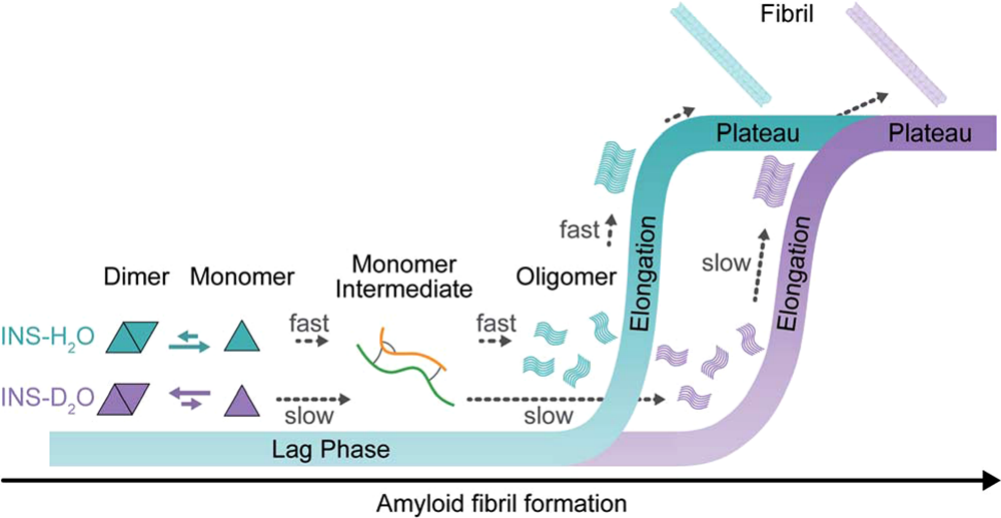
Insulin (INS) fibrillization kinetics in H_2_O and D_2_O and proposed fibrillization mechanism explaining the slower assembly in D_2_O. Reproduced from ref (39), Chun et al., published by the Royal Society of Chemistry (licensed under CC BY-NC 3.0).

Several studies have shown a significant difference in protein assembly rates in water and D_2_O, with assembly occurring *faster* in the latter. For instance, the aggregation and simultaneous double-helix formation of κ-carrageenan occurs faster in D_2_O than in H_2_O.^25^ Faster aggregation in D_2_O was also observed for gelatin,^37^ casein,^38^ and bovine serum albumin.^19^ These examples all show faster assembly in D_2_O, but self-assembly processes can also become *slower* in D_2_O. Recently, a ground-breaking study by Cho et al. has shown that amyloid formation of insulin occurs slower in D_2_O than in H_2_O (Figure 4).^39^ This effect was attributed to the presence of intermediates that adopt intermolecular beta-sheet structures, which are more favored in D_2_O than in H_2_O. Using D_2_O as a solvent instead of H_2_O increases the free-energy barrier for unfolding these intermediates, which is a necessary step for the final fibril formation. A similar enhancement of oligomer stability in heavy water was suggested for transthyretin tetramer.^49^ Interestingly, it was recently found that the fibrillization of alpha-synuclein (the protein responsible for Parkinson’s disease) proceeds *faster* in D_2_O than in water.^40^ This acceleration was attributed to enhanced protein–protein interactions in D_2_O that facilitate the refolding of alpha-synuclein, which is required for initiating its fibrillization.

Surprisingly, not only protein-assembly kinetics but even the viscoelastic properties of biopolymer networks can be different in D_2_O and H_2_O. The mechanical properties of reconstituted actin networks are affected by using D_2_O instead of H_2_O: in D_2_O the actin filaments behave as a transiently cross-linked network rather than the typical behavior of an entangled network (as is observed in H_2_O). This peculiar behavior in D_2_O was recently explained by the finding that D_2_O induces the formation of multifilament bundles, leading to a structural reorganization of the actin network and different mechanical properties.^52^ The difference in the network structure was attributed to a larger stickiness between actin filaments in D_2_O because of enhanced intermolecular interactions in this solvent.^14,52^ Similarly, the elastic modulus of gels formed by the aggregation of κ-carrageenan is ∼10–20% higher in D_2_O than in H_2_O because of the larger number of cross-links formed between the chains.^25^ Such increased network rigidity has also been observed in gelatin and casein gels.^37,38^ In contrast, Brenner et al. found that in agarose gel the mechanical properties are the same in D_2_O and H_2_O, even though D_2_O does enhance the stability of the helical structure and gives rise to gels with larger heterogeneity on the micrometer scale (and not the nanometer scale).^33^ Finally, an intriguing topological difference in biopolymer-network structure has been found in the case of pectin, for which recent experiments have shown that the fractal dimension of the gel network formed is higher in D_2_O than in H_2_O (indicating that in D_2_O the gel is more clustered),^41^ an observation that again “highlights the need to be mindful of changes induced when substituting D_2_O in systems with significant hydrogen bonding”.^41^

### The Origin of D_2_O-Induced Changes in Stability and Structure

In D_2_O, biopolymers are exposed to a more strongly hydrogen-bonded water network,^42^ and therefore creating a solvation cavity to accommodate the protein (or increasing the solvent-exposed surface area of a protein) is energetically less favorable in D_2_O because of the additional enthalpic cost required to break the water hydrogen bonds. This energetic loss is enhanced when the solvent needs to reorganize around nonpolar groups, and hence hydrophobic patches have a stronger tendency to cluster in D_2_O than in H_2_O, an effect we may refer to as isotopically enhanced hydrophobic effect. However, a theoretical analysis by Graziano and Pica has shown that the H_2_O/D_2_O effect on the hydrogen-bond structure may not be sufficient to explain D_2_O-enhanced protein stability.^11^ Due to the lower molecular polarizability of D_2_O, van der Waals attractive interactions are less favorable in D_2_O, and thus fewer interactions take place between protein and water. Reduced van der Waals interactions affect the binding affinity of D_2_O to biomacromolecules, which may lead to changes in the hydration shell surrounding the biomolecules.^23,35^ The combination of reduced van der Waals interaction and the higher enthalpic cost of water–water hydrogen-bond breaking will likely change the hydration capability of D_2_O with respect to H_2_O in a synergistic way. Since contacts between water and protein can reduce the free energy barrier between the different protein conformations, the lower number of water–protein interactions in D_2_O will lead to structurally more stable and less fluctuating proteins, as reported in the literature (Table 2). This proposed stabilization mechanism is also suggested in a recent study by Haidar et al.^53^ From collision-induced unfolding and ion-mobility mass spectrometry, it was found that the stability of lysozyme, cytochrome c, and bovine ubiquitin in the gas phase is independent of whether the protein is hydrogenated or fully deuterated, in contrast with the increased stability of these proteins in D_2_O solution, again indicating that the changes in protein properties are due to solvent effects. This idea seems to be further confirmed by the general absence of significant differences between the crystal structures of hydrogenated and perdeuterated proteins.^47,49^ A decrease in water–protein interaction in D_2_O compared to H_2_O is also consistent with the enhanced rigidity observed, for instance, in collagen peptides, where intramolecular hydrophobic interactions are minimal and thus enhanced hydrophobic effect alone cannot explain the increased rigidity.^24^

We have seen that biomolecular assembly can occur at different rates in D_2_O and H_2_O (Table 3 and Figure 4). If the aggregation is driven by hydrophobic or hydrophilic interactions, the kinetics are expected to be different in D_2_O. As discussed before, D_2_O enhances the hydrophobic interactions (enhancing the aggregation) and has reduced protein hydration compared to H_2_O. This latter effect implies that the desolvation enthalpy, i.e., the energy required to break the hydrogen bonds between water and hydrophilic groups to allow the formation of bonds between hydrophilic groups, is lower in D_2_O than in H_2_O. This is consistent with the faster assembly rate reported for several systems.^25,37,38^ However, if the aggregation process involves the formation of intermediates stabilized by hydrophobic interactions, the assembly might be slower in D_2_O, as observed in the case of amyloid formation.^39^ To form fibrils, intermediates have to undergo partial unfolding, a process that is energetically more unfavorable in D_2_O since the intermediates are more stable due to the enhanced hydrophobic effect.

## From Elephant in the Room to Opportunity for Protein Research

Although in general replacing H_2_O with D_2_O has a limited effect on protein structure (as is demonstrated by the large number of successful studies in which this procedure was used), the experiments and simulations discussed above show that replacing H_2_O with D_2_O can in some cases significantly change the structure and stability of proteins and protein assemblies. On the one hand, this means that experiments on proteins in which H_2_O has been replaced with D_2_O should be interpreted with caution. On the other hand, the possibility of “tuning” the hydration strength by varying the isotopic composition provides a unique tool to investigate protein hydration, and might be useful for gaining a better understanding of the role of water in defining protein structure. Water strongly influences the properties of proteins and is also believed to regulate and mediate protein–protein/ligand interactions in many biopolymers, such as collagen or silk fibroin, and water is also believed to play a crucial role in determining collagen interactions with minerals in bone tissue.^54^ Experiments designed to investigate protein hydration usually measure how the protein properties change upon varying the solvent, for instance, by replacing or mixing water with an organic solvent. This clearly changes the protein hydration but unfortunately also modifies many other solvent properties, such as the dielectric constant and the molecular size, which might affect protein intra- and intermolecular interactions. Replacing water with D_2_O is a unique method to specifically modify the water hydrogen bonding without changing the other solvent properties. Comparing protein behavior in H_2_O and D_2_O and their mixtures thus constitutes an elegant way to determine specifically the contribution of water hydrogen bonding to the physical and chemical properties of proteins without having to resort to changes in the solvent that alter more than the protein hydration. Such D_2_O vs H_2_O experiments may not always be easy to realize, but for instance two-dimensional infrared spectroscopy on proteins in H_2_O has already been reported.^39,55−57^ This recent advancement enables researchers to study proteins in more natural systems, such as in cells or in blood serum.^58,59^ Since the protein amide-I frequencies and line shapes may change upon H/D exchange, extracting structural information from such 2D-IR spectra in H_2_O will require adaptation of the currently existing theoretical and modeling framework, which was developed mainly for interpreting 2D-IR spectra of proteins in D_2_O; see ref. 58 for an excellent future perspective on this topic.

Since D_2_O enhances the hydrophobic effect, a comparison of protein secondary structure in H_2_O and D_2_O can reveal the role of hydrophobic interactions in the stabilization of the proteins or in promoting their collapse. Similarly, comparing self-assembly kinetics in water and D_2_O can be a valuable method to gain a better understanding of the aggregation process, in particular in the case of fibril formation. Fibril formation can occur spontaneously via a nucleation-and-growth mechanism (1-step-nucleation or 1SN) or in two steps via the formation of intermediate aggregates (2SN) stabilized by hydrophobic effects. Intermediates subsequently need to undergo structural transformations to attain the fibrillar conformation, representing the rate-limiting step for fibrillization. Since D_2_O stabilizes hydrophobic interactions, the aggregation rate in D_2_O with respect to H_2_O is reduced if the mechanism involves intermediates, because their unfolding is energetically more unfavorable in D_2_O. Comparing the fibrillization rate in water and D_2_O can therefore reveal whether intermediates are present and hence if the amyloid formation occurs by a 2SN or 1SN mechanism. On the same note, the ability of D_2_O to slow the aggregation and stabilize the intermediates can be used to study the intermediate species. Intermediates are transient and metastable aggregates, which are quite challenging to detect and characterize structurally. By using D_2_O, we can follow the protein self-assembly in “slow motion”.

Thus, we believe that the difference in biopolymer hydration in H_2_O and D_2_O can be exploited to gain a better understanding of biopolymers, in particular, of biopolymer–solvent interactions and their role in defining the structure and dynamics of proteins and protein assemblies. This constitutes an interesting next challenge for the scientific community working on proteins and protein assemblies.

## Footnotes

a We will use the term “hydrogen bond” to denote both hydrogen bonds and deuterium bonds.
